# Supplementary material for: Novel on-site follow-up and enhancement program (FEP) improves knowledge, clinical skills and enabling environment of skilled birth attendants in Nepal
Source: PLoS One. 2023 Aug 22;18(8):e0285653. doi: 10.1371/journal.pone.0285653 (PMC10443845; doi:10.1371/journal.pone.0285653)
Supplement: S2 Checklist — (DOCX) [file pone.0285653.s002.docx]

**Revised Standards for Quality Improvement Reporting Excellence (SQUIRE 2.0) September 15, 2015**

| **Text Section and Item**  **Name** | **Section or Item Description** |
| --- | --- |
| **Notes to authors** | - The SQUIRE guidelines provide a framework for reporting new   knowledge about how to improve healthcare   - The SQUIRE guidelines are intended for reports that describe [system](#_bookmark13) level work to improve the quality, safety, and value of healthcare, and used methods to establish that observed outcomes were due to the [intervention(s).](#_bookmark8) - A range of approaches exists for improving healthcare. SQUIRE may be adapted for reporting any of these. - Authors should consider every SQUIRE item, but it may be inappropriate or unnecessary to include every SQUIRE element in a particular manuscript. - The SQUIRE Glossary contains definitions of many of the key words in SQUIRE. - The Explanation and Elaboration document provides specific examples of well-written SQUIRE items, and an in-depth explanation of each item. - Please cite SQUIRE when it is used to write a manuscript. |
| **Title and Abstract** |  |
| **1. Title** | Novel on-site follow-up and enhancement program (FEP) improves knowledge, clinical skills and enabling environment of skilled birth attendants in Nepal |
| **2. Abstract** | **Introduction:** Although great strides have been made in maternal and newborn health in Nepal, the maternal mortality ratio (MMR) is still high at 186 per 100,000 births. Many maternal deaths are preventable if there is access to a skilled birth attendant (SBA).  The Ministry of Health and Population of Nepal launched the in-service SBA training program in 2007 and has trained over 10,000 SBAs to date. Evidence shows that one episode of training is not enough to retain skills. Therefore, the Nick Simons Institute (NSI) in collaboration with National Health Training Center (NHTC) devised a Follow-Up and Enhancement Program (FEP) in 2011 where the knowledge, clinical skills, and working environment of SBA graduates were assessed directly at their worksites. FEP allows on-site coaching and feedback so that graduates may continue to improve upon any gaps in their knowledge, skills, and working environment.  This study aims to assess the effectiveness of FEP.  **Methods:** We used a mixed-methods research design. A total of 73 SBAs who had a pre-FEP assessment in 2016 were followed up for a post-FEP assessment in 2017. We also collected data from 3 additional districts (115 SBAs) that had not previously had FEP, to compare SBAs in FEP versus non-FEP districts. Qualitative data was collected from 16 health facilities on the perceptions, motivation, and satisfaction of stakeholders.  **Results:** Of the total 188 SBAs that were sampled, a one-time FEP increased knowledge scores by 9%, clinical skills scores by 29%, and enabling environment scores by 7%. The number of deliveries conducted improved with a one-time FEP, although this increase was not statistically significant. We found a trickle-down effect of working in a facility that has had prior FEP, with SBAs that have never had FEP improving their clinical skills. FEP was found to be a highly accepted program and is beneficial to SBAs, trainers, and the Hospital Management Committee (HFOMC). However, a one-time FEP is not sufficient in retaining clinical skills and knowledge.  **Conclusion:** FEP is a highly effective program by both quantitative and qualitative evaluation. Our study suggests that FEP should be frequent and continuous to retain the knowledge and clinical skills of SBAs, motivate them through on-site coaching, and improve their working environment through direct feedback to the Ministry of Health and Population. |
| **Introduction** | *Why did you start?* |
| [**3. Problem**](#_bookmark10) [**Description**](#_bookmark10) | Nature and significance of the local [problem](#_bookmark10)  Although great strides have been made in maternal and newborn health in Nepal, the maternal mortality ratio (MMR) is still high at 186 per 100,000 births [1].  The rate of decline in the MMR has slowed down significantly despite increased utilization of maternal and neonatal health (MNH) services and visits.  For example, between 2006 and 2016, institutional delivery by a skilled birth attendant (SBA) increased from 18 to 59% while the MMR only decreased from 281 to 259 per 100,000 [2].  In addition, substantial disparities exist in access to MNH services, with the urban population with higher socio-economic status benefiting while leaving poor and rural communities behind [3 -5]. One factor for the slow improvement in MMR and in-country disparities is the actual quality of care being provided to mothers and newborns.  Evidence suggests the MMR is highest among disadvantaged groups, who have poorer coverage of routine MNH visits and receive poorer quality of care during those routine visits [2].  Furthermore, our prior study showed that while countries like Nepal have made important investments in SBA programs, these healthcare workers are failing to receive either effective training or sufficient practice to stay clinically competent and knowledgeable in the field [6,7].  In order to address issues of the quality of skilled birth attendants and their training, in 2011, the National Health Training Center (NHTC) in collaboration with the Nick Simons Institute (NSI) devised a novel program called the Follow-Up Enhancement Program (FEP). The goal of FEP was to cultivate a pioneer program of supportive supervision where the clinical knowledge and skills of SBAs who have received in-service training are assessed *directly at their working sites* [8]. The objectives of FEP are to assess the knowledge and skills of SBAs after training, explore the reasons for skill and knowledge weaknesses, determine how much practical experience SBAs have in conducting deliveries and key maternity procedures, describe what factors affect the ability of SBAs to implement tools acquired in training and provide future recommendations. In addition, the FEP allows *on-site* coaching and feedback so that trainees may continue to improve upon any gaps in their skills and knowledge. FEP furthermore includes an assessment of the trainees’ working environment in terms of essential equipment, drug supply, and team support, collectively termed the Enabling Environment Score. A good enabling environment is crucial to overcoming poor service utilization or poor service provision for quality maternal and neonatal health services [9, 10]. The clinical skills, knowledge, and enabling environment assessments from FEP are then fed back to the Health Facility Operation and Management Committees (HFOMC) at district and central levels so that gaps can be fulfilled [8]. |
| **4. Available knowledge** | Summary of what is currently known about the [problem,](#_bookmark10) including relevant previous studies  Although great strides have been made in maternal and newborn health in Nepal, the maternal mortality ratio (MMR) is still high at 186 per 100,000 births [1].  The rate of decline in the MMR has slowed down significantly despite increased utilization of maternal and neonatal health (MNH) services and visits.  For example, between 2006 and 2016, institutional delivery by a skilled birth attendant (SBA) increased from 18 to 59% while the MMR only decreased from 281 to 259 per 100,000 [2].  In addition, substantial disparities exist in access to MNH services, with the urban population with higher socio-economic status benefiting while leaving poor and rural communities behind [3 -5]. One factor for the slow improvement in MMR and in-country disparities is the actual quality of care being provided to mothers and newborns.  Evidence suggests the MMR is highest among disadvantaged groups, who have poorer coverage of routine MNH visits and receive poorer quality of care during those routine visits [2].  Furthermore, our prior study showed that while countries like Nepal have made important investments in SBA programs, these healthcare workers are failing to receive either effective training or sufficient practice to stay clinically competent and knowledgeable in the field [6,7]. |

| **5.** [**Rationale**](#_bookmark12) | Informal or formal frameworks, models, concepts, and/or [theories](#_bookmark14) used to explain the [problem,](#_bookmark10) any reasons or [assumptions](#_bookmark0) that were used to develop the [intervention(s),](#_bookmark8) and reasons why the [intervention(s)](#_bookmark8) was  expected to work  Although great strides have been made in maternal and newborn health in Nepal, the maternal mortality ratio (MMR) is still high at 186 per 100,000 births [1].  The rate of decline in the MMR has slowed down significantly despite increased utilization of maternal and neonatal health (MNH) services and visits.  For example, between 2006 and 2016, institutional delivery by a skilled birth attendant (SBA) increased from 18 to 59% while the MMR only decreased from 281 to 259 per 100,000 [2].  In addition, substantial disparities exist in access to MNH services, with the urban population with higher socio-economic status benefiting while leaving poor and rural communities behind [3 -5]. One factor for the slow improvement in MMR and in-country disparities is the actual quality of care being provided to mothers and newborns.  Evidence suggests the MMR is highest among disadvantaged groups, who have poorer coverage of routine MNH visits and receive poorer quality of care during those routine visits [2].  Furthermore, our prior study showed that while countries like Nepal have made important investments in SBA programs, these healthcare workers are failing to receive either effective training or sufficient practice to stay clinically competent and knowledgeable in the field [6,7].  In order to address issues of the quality of skilled birth attendants and their training, in 2011, the National Health Training Center (NHTC) in collaboration with the Nick Simons Institute (NSI) devised a novel program called the Follow-Up Enhancement Program (FEP). The goal of FEP was to cultivate a pioneer program of supportive supervision where the clinical knowledge and skills of SBAs who have received in-service training are assessed *directly at their working sites* [8]. The objectives of FEP are to assess the knowledge and skills of SBAs after training, explore the reasons for skill and knowledge weaknesses, determine how much practical experience SBAs have in conducting deliveries and key maternity procedures, describe what factors affect the ability of SBAs to implement tools acquired in training and provide future recommendations. In addition, the FEP allows *on-site* coaching and feedback so that trainees may continue to improve upon any gaps in their skills and knowledge. FEP furthermore includes an assessment of the trainees’ working environment in terms of essential equipment, drug supply, and team support, collectively termed the Enabling Environment Score. A good enabling environment is crucial to overcoming poor service utilization or poor service provision for quality maternal and neonatal health services [9, 10]. The clinical skills, knowledge, and enabling environment assessments from FEP are then fed back to the Health Facility Operation and Management Committees (HFOMC) at district and central levels so that gaps can be fulfilled [8].  One of the main components used for the assessment is a paper FEP tool (see Appendix), administered to individual SBAs by SBA trainers, who are doctors and nurses who have completed an SBA course, Clinical Training Skills course, and National Health Training Center certification, and have 1–4 years of SBA training experience, with the assistance of NSI and public health nurses. The SBA FEP tool was drafted by SBA trainers, Support for Safer Motherhood Project, and the Family Health Division and was pretested in five districts, the findings of which were disseminated to stakeholders and used to revise the tool. The assessment, which included background information, knowledge and clinical assessment, clinical decision-making, infection prevention, and an enabling environment checklist, took approximately 5 hours for each SBA to complete [6, 8].  As of 2021, NSI and NHTC had implemented FEP in 47 districts throughout Nepal, visiting 762  birthing centers with a follow-up of  1408 SBAs [11, 12]. However, to date, there has not been a study to assess the actual effectiveness of FEP. This study, therefore, aims to:   1. Assess the impact of FEP on the knowledge and clinical skills of SBAs and the enabling environment of the health facilities in which they work 2. Assess the impact of FEP on the number of deliveries being conducted by SBAs 3. Assess stakeholder perceptions regarding FEP delivery, design, implementation, and follow-up |
| --- | --- |
| **6. Specific aims** | This study aims to:   - Assess the impact of FEP on the knowledge and clinical skills of SBAs and the enabling environment of the health facilities in which they work - Assess the impact of FEP on the number of deliveries being conducted by SBAs - Assess stakeholder perceptions regarding FEP delivery, design, implementation, and follow-up |
| **Methods** | *What did you do?* |
| **7.** [**Context**](#_bookmark1) | Although great strides have been made in maternal and newborn health in Nepal, the maternal mortality ratio (MMR) is still high at 186 per 100,000 births [1].  The rate of decline in the MMR has slowed down significantly despite increased utilization of maternal and neonatal health (MNH) services and visits.  For example, between 2006 and 2016, institutional delivery by a skilled birth attendant (SBA) increased from 18 to 59% while the MMR only decreased from 281 to 259 per 100,000 [2].  In addition, substantial disparities exist in access to MNH services, with the urban population with higher socio-economic status benefiting while leaving poor and rural communities behind [3 -5]. One factor for the slow improvement in MMR and in-country disparities is the actual quality of care being provided to mothers and newborns.  Evidence suggests the MMR is highest among disadvantaged groups, who have poorer coverage of routine MNH visits and receive poorer quality of care during those routine visits [2].  Furthermore, our prior study showed that while countries like Nepal have made important investments in SBA programs, these healthcare workers are failing to receive either effective training or sufficient practice to stay clinically competent and knowledgeable in the field [6,7]. |
| **8.** [**Intervention(s)**](#_bookmark8) | 1. Description of the [intervention(s)](#_bookmark8) in sufficient detail that others could reproduce it 2. Specifics of the team involved in the work   In order to address issues of the quality of skilled birth attendants and their training, in 2011, the National Health Training Center (NHTC) in collaboration with the Nick Simons Institute (NSI) devised a novel program called the Follow-Up Enhancement Program (FEP). The goal of FEP was to cultivate a pioneer program of supportive supervision where the clinical knowledge and skills of SBAs who have received in-service training are assessed *directly at their working sites* [8]. The objectives of FEP are to assess the knowledge and skills of SBAs after training, explore the reasons for skill and knowledge weaknesses, determine how much practical experience SBAs have in conducting deliveries and key maternity procedures, describe what factors affect the ability of SBAs to implement tools acquired in training and provide future recommendations. In addition, the FEP allows *on-site* coaching and feedback so that trainees may continue to improve upon any gaps in their skills and knowledge. FEP furthermore includes an assessment of the trainees’ working environment in terms of essential equipment, drug supply, and team support, collectively termed the Enabling Environment Score. A good enabling environment is crucial to overcoming poor service utilization or poor service provision for quality maternal and neonatal health services [9, 10]. The clinical skills, knowledge, and enabling environment assessments from FEP are then fed back to the Health Facility Operation and Management Committees (HFOMC) at district and central levels so that gaps can be fulfilled [8]. |
| **9. Study of the Intervention(s)** | 1. Approach chosen for assessing the impact of the [intervention(s)](#_bookmark8) 2. Approach used to establish whether the observed outcomes were due to the [intervention(s)](#_bookmark8)   We used a convergent mixed method approach whereby we triangulated qualitative and quantitative data. For the quantitative component, the clinical knowledge and skills and enabling environment of  73 SBAs working in  birthing centers who had undergone FEP as well as  93 SBAs working in  birthing centers who had never had FEP were assessed.  All the SBAs included in the study were nurses (auxiliary nurse midwife or staff nurse). Furthermore, we assessed clinical knowledge and skills of 22 SBAs working in a facility with other SBAs that had previously undergone FEP.  Four external consultants who were SBA trainers were hired to assess the clinical knowledge, skills and enabling environment Qualitative data were collected simultaneously using semi-structured interviews and focus group discussions from 16 birthing centers in 3 districts (Bardiya, Makawanpur, Bajhang) where SBAs received FEP intervention.  Semi-structured interviews were conducted with 30 SBAs, and 3 District Health Officers (DHO), 3 Public Health Nurses(PHN) and 1 central level stakeholder. Focus group discussions were conducted with 16 HFOMC members and 2 focus group discussions were conducted with SBA trainers at 2 training sites. There were 6 to 8 people in each focus group discussion, and discussions took up to 45- 90 minutes in duration. |
| **10. Measures** | We used a convergent mixed method approach whereby we triangulated qualitative and quantitative data. For the quantitative component, the clinical knowledge and skills and enabling environment of  73 SBAs working in  birthing centers who had undergone FEP as well as  93 SBAs working in  birthing centers who had never had FEP were assessed.  All the SBAs included in the study were nurses (auxiliary nurse midwife or staff nurse). Furthermore, we assessed clinical knowledge and skills of 22 SBAs working in a facility with other SBAs that had previously undergone FEP.  Four external consultants who were SBA trainers were hired to assess the clinical knowledge, skills and enabling environment. The knowledge of procedures and managing complications was assessed using a 20-question multiple-choice survey [68]. The clinical competence level was evaluated using standardized checklists on anatomical models and clinical case vignettes for the use of partograph, normal and vacuum delivery, newborn resuscitation, referral, and care of pregnancy complications [68].  The enabling environment was assessed by a FEP trainer using a thorough checklist that included each facility’s equipment, infrastructure, and medications [68]. Furthermore, the total monthly deliveries for each SBA was assessed by reviewing each facility’s delivery logs, and information on the number of normal deliveries, vacuum deliveries, breech deliveries, and the management of any of the following seven complications over a 3- month period were recorded. These complications included postpartum hemorrhage, antepartum hemorrhage, eclampsia, episiotomy, manual placenta removal, post-abortion care, and newborn resuscitation [68]. The statistical significance level was set at a p-value threshold of 0.05, indicating that any result with p-values less than 0.05 was considered statistically significant.  Qualitative data were collected simultaneously using semi-structured interviews and focus group discussions from 16 birthing centers in 3 districts (Bardiya, Makawanpur, Bajhang) where SBAs received FEP intervention.  Semi-structured interviews were conducted with 30 SBAs, and 3 District Health Officers (DHO), 3 Public Health Nurses(PHN) and 1 central level stakeholder. Focus group discussions were conducted with 16 HFOMC members and 2 focus group discussions were conducted with SBA trainers at 2 training sites. There were 6 to 8 people in each focus group discussion, and discussions took up to 45- 90 minutes in duration.  Topic guides were developed in an iterative process throughout data collection to guide the interviews and discussions.  An external team of trained Nepali field researchers collected qualitative data. They were oriented to the purpose of study and topic guides for 2 days prior to field visits. Participants gave written informed consent to participate. All the discussions were digitally recorded in the local Nepali language and transcribed and translated into English. 5 pages of 3 randomly selected transcripts were back translated into Nepali and compared with recordings to check the quality of translation. |
| **11. Analysis** | Quantitative data was collected in paper form and entered into Stata and analyzed.  Participants were assigned identification numbers and data was made anonymous. The pre-post analysis was done to assess if there is a difference between the same SBAs a year after they undergo FEP in terms of knowledge, clinical skills, and the number of deliveries and whether there was a change in their enabling environment. The intergroup comparative analysis was done to assess any difference between SBAs that had a one-time FEP and those who never had it, and any trickle-down effect of working in a FEP facility (Fig 3). For qualitative data, we conducted a thematic content analysis, whereby the  research team read a sample of the transcripts. A descriptive report of the preliminary analysis was written by researchers after which the research team independently generated themes from the data and came to a consensus through discussion. The data were coded according to the themes in NVivo 12. |
| **12. Ethical**  **Considerations** | [Ethical aspects](#_bookmark2) of implementing and studying the [intervention(s)](#_bookmark8) and how they were addressed, including, but not limited to, formal ethics review and potential conflict(s) of interest  Ethical approval was obtained from the Nepal Health Research Council (Registration number: 415/2017). for the data collection and questionnaire design. The investigators sought written informed consent before enrolling study participants. The investigators provided information about the study’s objective to the study participants using the local language. The participants were assured complete confidentiality. The datasets generated during and/or analyzed during the current study are available from the corresponding author upon reasonable request.  The datasets that were used in this study have sensitive information such as the personal information of the participants. Additionally, the dataset is owned and maintained by the Ministry of Health and Population of Nepal  and NSI, and there are restrictions on publicly sharing data to protect participants' privacy. However, interested parties can obtain the data on which this study was based by submitting a request to NSI which will then initiate the process of obtaining formal approval through the MoHP on a case-by-case basis. Data requests can be sent to the Research Unit at NSI by emailing suresh_tamang@nsi.edu.np.  Our study was supported by the Nick Simons Institute. There is no other conflict of interest to report. |
| **Results** | *What did you find?* |
| **13. Results** | Quantitative FindingsPre-Post Analysis A total of 188 SBAs were sampled. 73 SBAs were still present at the facility where prior FEP had been conducted and underwent pre- and post- FEP analysis. Another 22 SBAs were working at the same facility where prior FEP had been conducted and were also assessed. 93 SBAs were from 3 districts where FEP had not been conducted. SBAs on average exhibited an improvement in knowledge, clinical skills, enabling environment, and total deliveries following a one-time FEP. As seen in Table 1A, of the total 73 SBAs still present at the facility where FEP had been conducted, total knowledge scores improved with one-time FEP by approximately 9%. SBAs who received FEP achieved a total mean score of 84% on the written knowledge assessment compared to the 75% total mean score achieved by SBAs before receiving FEP (p<0.001). After receiving FEP, SBAs on average achieved 8.22% higher on partograph knowledge (p=0.03), 10.96% higher on PPH knowledge (p=0.16), 14.38% higher on infection prevention knowledge (p<0.001), and 18.72% higher on vacuum delivery knowledge (p<0.001).  Total clinical skills scores improved with one-time FEP by approximately 29%. SBAs who received FEP achieved a total mean score of 75% on the clinical skills assessment compared to the 46% total mean score achieved by SBAs before receiving FEP (p<0.001). After receiving FEP, SBAs on average achieved 32.19% higher in the management of eclampsia (p<0.001) and 37.75% higher in the management of shock (p<0.001). However, there was a 3.03% decrease in vacuum delivery skills following a one-time FEP, but this was not found to be statistically significant (p=0.48).  An examination of the total enabling environment scores revealed that a one-time FEP improved the enabling environment scores by approximately 7%. SBAs who received FEP achieved a total mean score of 78% on the enabling environment assessment compared to the 71% total mean score achieved by SBAs before receiving FEP (p=0.09). Upon analysis, the components of the enabling environment score that improved significantly with one-time FEP were labor equipment, general equipment, supplies, and team support. .  In addition, as seen in Table 1B, we also assessed whether there was a difference in the number of deliveries conducted by SBAs pre-and post-FEP. After receiving FEP, SBAs conducted 4.7 additional total deliveries (mostly normal deliveries) over 3 months, although this increase was not found to be statistically significant (p=0.17). There was an increase of 4.76 normal deliveries (p=0.15), an increase of 0.02 breech deliveries (p=0.79), and a decrease of 0.1 vacuum deliveries (p=0.39) following a one-time FEP, but these were not found to be statistically significant. Intergroup Comparative Analysis Table 2 examines if there is any difference between 73 SBAs that underwent FEP compared to 93 SBAs that had not yet had FEP in 2017. The SBAs that had already undergone FEP had total knowledge scores of approximately 84% compared to the 80% total knowledge scores achieved by the SBAs that were yet to undergo FEP (p=0.04). In terms of clinical skills, the SBAs that had undergone FEP achieved total scores of 75% compared to 41% scored by those that were yet to undergo FEP (p<0.001). Additionally, there is an observed difference in the enabling environment of SBAs that underwent FEP compared to the SBAs that had yet to undergo FEP. Those that already underwent FEP achieved total enabling environment scores of 77% compared to the 57% scored by those that were yet to undergo FEP (p<0.001). In terms of total deliveries, there is a 5.46 increase in deliveries over 3 months for the 73 SBAs that underwent FEP compared to the 93 SBAs that were yet to undergo FEP. This difference, however, is not found to be statistically significant (p=0.10).  Table 3 examines the effect of working at a site that had previously undergone FEP. It compares 93 SBAs who had not yet undergone FEP in 2017 with 22 SBAs who had also never undergone FEP but were working at a site where FEP was previously conducted. SBAs working at a prior FEP site had total knowledge scores of approximately 87% compared to 80% total knowledge scores achieved by SBAs not working at a prior FEP site (p=0.01).  There is an observed difference in clinical skills with SBAs working at a prior FEP facility having skills scores of 81% compared to 41% for those not working at a prior FEP site (p<0.001). This is also true in the observed difference in enabling environment with SBAs working at a prior FEP site achieving enabling environment scores of 83% compared to 58% for those not working at a prior FEP site (p<0.001). In terms of total deliveries, SBAs working at a prior FEP site conducted 7.88 additional deliveries over 3 months compared to SBAs that worked at non-FEP sites. However, this difference was not found to be statistically significant (p=0.39). Qualitative Findings **Positive Perceptions of FEP**  Our qualitative interviews and data found that FEP is an ideal monitoring and support program that is highly accepted and appreciated (Fig 4). It was found that the FEP is beneficial to all stakeholders involved—SBAs, trainers, and the HFOMC. FEP updates knowledge and skills, energizes and builds confidence, and establishes linkages between SBA trainers and HFOMC, with one SBA from mountain district (Bajhang) stating, “*It’s been a long time since we took SBA training. We may forget. Patient flow is low here. That is why it [FEP] refreshes us. We can revise whatever we have forgotten. We can correct our mistakes.”* Additionally, SBAs felt that FEP enhanced their ability to conduct deliveries according to protocol. In the words of one SBA working in the birthing center of the Plain area (Bardiya) *“Now we can successfully conduct deliveries using the methods of infection prevention in a skilled way. We used to do deliveries in a haphazard way prior to the [FEP] training but after the training, we are doing deliveries in a systematic way.”*  FEP also updates knowledge and skills, provides an opportunity to see real-world scenarios, and establishes linkages between the different stakeholders involved, with one trainer from Bharatpur recounting, *“Providing messages only is not enough. If we go to the FEP, then we can understand how the SBA trainees are doing* ***at their sites of work****, whether they are doing what we taught them or not.”* FEP is beneficial to the HFOMC because they become aware of what their responsibilities are. It energizes the HFOMC and establishes a linkage between the HFOMC and SBAs, with one HFOMC member in Hill area (Makawanpur) reporting, “*Communication is frequently made with the staff. We are also aware of the various aspects of their work so that a better working environment can be created, and maximum service can be provided. We focus our concentration on making things easier for the staff.”*  **Improvements Needed in FEP**  Although FEP was generally well received, informants offered several suggestions to improve the program (Fig 5). Most participants wished that FEP occurred at more regular time intervals, with every six to twelve months as an ideal timeframe. In addition, more frequent intervals would also keep the health management committee more responsible for completing their tasks. One member of the HFOMC of the Plain area (Bardiya) stated, *“If you visited our organization every six months...we can remember. We cannot remember about the activities which were conducted 1.5 years ago.”* In addition, participants generally would have preferred FEP to occur over a few days with a shorter working day, with one SBA of Hill area (Makawanpur) lamenting, *“This one-day duration is too little, and we have to do everything in a hurry.”*  Some respondents wanted to receive equipment and incentives after participating in FEP. For instance, one member of the HFOMC from the Plain area (Bardiya) said, “*As they come here and observe and tell us the things we need to improve, but it would be good if they manage (help us get or give us) materials too.”* In terms of the training, participants also wanted more educational materials, training during live deliveries, training more applicable to the environment of uncomplicated deliveries, and in some cases better teaching methods. Besides providing educational materials in person, a few participants mentioned sending updated materials throughout the year. |
| **Discussion** | *What does it mean?* |
| **14. Summary** | 1. Key findings, including relevance to the [rationale](#_bookmark12) and specific aims 2. Particular strengths of the project   Our study evaluated the effect of FEP on the knowledge, clinical skills, enabling environment, and number of deliveries of SBAs in Nepal. We found that a one-time FEP significantly increases the knowledge and clinical skills scores of SBAs. Additionally, we found that a one-time FEP significantly improves the enabling environment of the health facilities in which the SBAs work. There was also an improvement in the delivery volume conducted by SBAs following a one-time FEP, although this was not statistically significant. Our study found that there is even a trickle-down effect of working in a facility that has had prior FEP, with SBAs that have never undergone FEP showing improved clinical skills.  The improvement in knowledge and clinical skills through an on-site mentoring program like FEP has been confirmed in other studies. A study conducted in Karnataka, India from 2012-2014 implemented a nurse mentor program at PHCCs to improve quality services [13]. Using a similar evaluation tool, the study found that the nurse mentoring program improved the staff’s knowledge and skills. Similar to our study, the checklist evaluation tool implemented in this study was also used to improve the working environment in terms of infrastructure, equipment, and drug supply. However, this study did not analyze any quantitative data and the results were all qualitative. A more recent study conducted in Nepal between 2016-2018 assessed an on-site clinical mentoring program and its effect on mentees’ knowledge and skills [14]. Of the 308 nurses assessed, clinical skills scores increased significantly, with SBA-trained mentees having better knowledge scores and performance on the 12 core clinical skills assessed by the program. There has been one randomized controlled trial also in Karnataka, India that used a checklist evaluation tool in conjunction with on-site nurse mentoring as the study intervention [15]. This study found that on-site mentoring, used with a checklist evaluation tool, improved knowledge, and skills by 2.3 times, compared to just a checklist evaluation tool alone, further supporting the value of hands-on mentoring.  In addition, both the qualitative and quantitative data suggested that the FEP program establishes communication linkages between the HFOMC and the SBAs, which is crucial for improving the enabling environment. One SBA working in the birthing center of Bajhang district stated, *“During FEP feedback, we discussed and met with the management committee. Now, it (HFOMC) has become active. They came to know their responsibilities. They listen to us and fulfill the essential materials we need”.* A study conducted in Nepal between 2014 and 2015 found that the HFOMC’s role was confined to mobilizing additional resources for the health facility, supporting infrastructure, and raising health awareness [16]. There was limited communication between the HFOMC members and the SBAs. There is a strong need for a program that establishes linkages between the HFOMC and SBAs and other staff at health facilities so that they can be involved in health needs assessment, planning, staffing, and fund management to promote a positive enabling environment at the health facility.  Many of the SBAs and other stakeholders involved in FEP reported that a one-time FEP was not enough and that it must be carried out with greater frequency. This need for continuous mentoring has been supported by other studies including a study conducted in Bihar, India that evaluated 319 public PHCCs and compared the intrapartum quality of care between 179 mentored and 80 non-mentored PHCCs [17]. This study found that the task completion scores were higher among mentored nurses but plateaued after one year, suggesting that mentoring needs to be ongoing. Other studies, such as one conducted in Nepal, looked at 104 SBAs and found that SBAs that underwent repeat training demonstrated greater clinical skills than those who did not [18].  The investigation boasts several strengths, including a mixed methods research design that integrates both quantitative and qualitative research methods to provide a more comprehensive understanding of the effectiveness of the Follow-Up Enhancement Program (FEP). The quantitative section of the study is strong in that it includes not only a pre-post analysis but also a comparative analysis of data from three additional districts that had not previously had FEP, enabling a comparison of skilled birth attendants (SBAs) in FEP versus non-FEP districts. Importantly, the study draws actionable conclusions that recommend frequent and continuous FEP to retain the knowledge and clinical skills of SBAs, motivate them through on-site coaching, and improve their working environment through direct feedback to the Ministry of Health and Population. These conclusions provide clear recommendations for policymakers and practitioners to improve maternal and neonatal health in Nepal. |

| **15. Interpretation** | 1. Nature of the association between the [intervention(s)](#_bookmark8) and the outcomes 2. Comparison of results with findings from other publications 3. Impact of the project on people and [systems](#_bookmark13) 4. Reasons for any differences between observed and anticipated outcomes, including the influence of [context](#_bookmark1) 5. Costs and strategic trade-offs, including [opportunity costs](#_bookmark9)   FEP is a highly effective program by both quantitative and qualitative evaluation. A one-time FEP significantly increases the knowledge and clinical skills scores of SBAs as well as the enabling environment of the health facilities in which they work. Our study suggests that FEP should be frequent and continuous. Continuation and scaling up of the FEP program are vital to retaining the knowledge and clinical skills of SBAs, motivating them through on-site coaching, and improving the working environment through direct feedback to the MoHP. FEP allows for an intimate understanding of the local working environment of SBAs and thereby leads to more effective health policies. |
| --- | --- |
| **16. Limitations** | 1. Limits to the [generalizability](#_bookmark3) of the work 2. Factors that might have limited [internal validity](#_bookmark7) such as confounding, bias, or imprecision in the design, methods, measurement, or analysis 3. Efforts made to minimize and adjust for limitations   Though we sampled a total of 188 SBAs for our study, this is a relatively small sample size which may affect generalizability. Moreover, we assessed SBAs only in Nepal, and the results may be specific to this region which could further impede generalizability. As with any observational study, we cannot determine causality. Residual confounding could also be present even after controlling for other factors. |
| **17. Conclusions** | 1. Usefulness of the work 2. Sustainability 3. Potential for spread to other [contexts](#_bookmark1) 4. Implications for practice and for further study in the field 5. Suggested next steps   Overall, FEP is a highly effective program by both quantitative and qualitative evaluation. A one-time FEP significantly increases the knowledge and clinical skills scores of SBAs as well as the enabling environment of the health facilities in which they work. Our study suggests that FEP should be frequent and continuous. Continuation and scaling up of the FEP program are vital to retaining the knowledge and clinical skills of SBAs, motivating them through on-site coaching, and improving the working environment through direct feedback to the MoHP. FEP allows for an intimate understanding of the local working environment of SBAs and thereby leads to more effective health policies. |
| **Other information** |  |
| **18. Funding** | Funding for the Follow-up and Enhancement Program, which carried out the data collection for the study in conjunction with the National Health Training Center (NHTC) of the Ministry of Health and Population of Nepal, came entirely from the Nick Simons Institute (http://www.nsi.edu.np/en), a charitable organization that works to train and support healthcare workers in rural Nepal (www.nsi.edu.np). The Nick Simons Institute initiated this study as part of its mission to continually improve care delivery in rural Nepal. Accordingly, it will use the study results to both raise awareness and lobby for changes in skilled birth attendant training, recruitment, and follow-up in Nepal and internationally. Neither the Nick Simons Institute nor the authors stand to receive material gain from the publication of this study. |

**Table 2. Glossary of key terms used in SQUIRE 2.0. This Glossary provides the intended meaning of selected words and phrases as they are used in the SQUIRE 2.0 Guidelines. They may, and often do, have different meanings in other disciplines, situations, and settings .**

**Assumptions**

Reasons for choosing the activities and tools used to bring about changes in healthcare services at the [system](#_bookmark13) level.

# Context

Physical and sociocultural makeup of the local environment (for example, external environmental factors, organizational dynamics, collaboration, resources, leadership, and the like), and the interpretation of these factors (“sense-making”) by the healthcare delivery professionals, patients, and caregivers that can affect the effectiveness and [generalizability](#_bookmark3) of [intervention(s).](#_bookmark8)

# Ethical aspects

The value of [system-](#_bookmark13)level [initiatives](#_bookmark6) relative to their potential for harm, burden, and cost to the stakeholders. Potential harms particularly associated with efforts to improve the quality, safety, and value of healthcare services include [opportunity costs,](#_bookmark9) invasion of privacy, and staff distress resulting from disclosure of poor performance.

# Generalizability

The likelihood that the [intervention(s)](#_bookmark8) in a particular report would produce similar results in other settings, situations, or environments (also referred to as external validity).

# Healthcare improvement

Any systematic effort intended to raise the quality, safety, and value of healthcare services, usually done at the [system](#_bookmark13) level. We encourage the use of this phrase rather than “quality improvement,” which often refers to more narrowly defined approaches.

# Inferences

The meaning of findings or data, as interpreted by the stakeholders in healthcare services –improvers, healthcare delivery professionals, and/or patients and families

# Initiative

A broad term that can refer to organization-wide programs, narrowly focused projects, or the details of specific interventions (for example, planning, execution, and assessment)

# Internal validity

Demonstrable, credible evidence for efficacy (meaningful impact or change) resulting from introduction of a specific intervention into a particular healthcare [system.](#_bookmark13)

# Intervention(s)

The specific activities and tools introduced into a healthcare [system](#_bookmark13) with the aim of changing its performance for the better. Complete description of an intervention includes its inputs, internal activities, and outputs (in the form of a logic model, for example), and the mechanism(s) by which these components are expected to produce changes in a [system’s](#_bookmark13) performance.

# Opportunity costs

Loss of the ability to perform other tasks or meet other responsibilities resulting from the diversion of resources needed to introduce, test, or sustain a particular [improvement](#_bookmark4) initiative

# Problem

Meaningful disruption, failure, inadequacy, distress, confusion or other dysfunction in a healthcare service delivery [system](#_bookmark13) that adversely affects patients, staff, or the [system](#_bookmark13) as a whole, or that prevents care from reaching its full potential

# Process

The routines and other activities through which healthcare services are delivered

# Rationale

Explanation of why particular [intervention(s)](#_bookmark8) were chosen and why it was expected to work, be sustainable, and be replicable elsewhere.

# Systems

The interrelated structures, people, [processes,](#_bookmark11) and activities that together create healthcare services for and with individual patients and populations. For example, systems exist from the personal self-care system of a patient, to the individual provider-patient dyad system, to the microsystem, to the macrosystem, and all the way to the market/social/insurance system. These levels are nested within each other.

# Theory or theories

Any “reason-giving” account that asserts causal relationships between variables (causal theory) or that makes sense of an otherwise obscure [process](#_bookmark11) or situation (explanatory theory). Theories come in many forms, and serve different purposes in the phases of [improvement](#_bookmark4) work. It is important to be explicit and well-founded about any informal and formal theory (or theories) that are used.
